# The health production function in South Asia: panel evidence on education, health expenditure, governance, and longevity

**DOI:** 10.3389/fpubh.2026.1866937

**Published:** 2026-09-16

**Authors:** Mohammad Ridwan, Zulfiquar Ali Antor, Jeremy Ko, Md. Emran Hossain, Chun Kai Leung, Wai-Kit Ming, Mohd Ziaur Rehman

**Affiliations:** 1 Department of Economics, Noakhali Science and Technology University, Noakhali, Bangladesh; 2 ARUCAD Research Centre, Arkin University of Creative Arts and Design, Mersin, Türkiye; 3 Centre for Comparative and International Studies, ETH Zurich, Zürich, Switzerland; 4 Erasmus School of Social and Behavioural Sciences, Erasmus University Rotterdam, Rotterdam, Netherlands; 5 Advanced Research Centre, European University of Lefke, Lefke, Northern Cyprus, Mersin, Türkiye; 6 Jadara Research Center, Jadara University, Irbid, Jordan; 7 Global Society and Sustainability Lab, University of Hong Kong, Hong Kong, Hong Kong SAR, China; 8 Department of Infectious Diseases and Public Health, City University of Hong Kong, Kowloon, Hong Kong SAR, China; 9 Department of Finance, College of Business Administration, King Saud University, Riyadh, Saudi Arabia

**Keywords:** education, government effectiveness, health expenditure, life expectancy, urbanization

## Abstract

This study examines the association of education, government effectiveness, health expenditure, economic growth, and urbanization with life expectancy in South Asia within Grossman’s health capital framework. Using annual panel data for seven South Asian countries—Bangladesh, Bhutan, India, Maldives, Nepal, Pakistan, and Sri Lanka—over 2000–2025, the analysis investigates both long-run and short-run relationships. The empirical strategy combines panel unit root tests, panel ARDL estimation, Driscoll–Kraay standard errors, PCSE, FGLS, System GMM, and bootstrap panel causality tests. The results indicate a stable long-run relationship among the variables. Education, measured by school enrollment and adult literacy, is positively and significantly associated with life expectancy. GDP per capita also shows a positive and significant association. Health expenditure has a positive but model-sensitive association, suggesting that the effectiveness of spending may matter more than its level alone. By contrast, government effectiveness and urbanization are significantly associated with life expectancy but with negative coefficients in the estimated models. Bootstrap causality results indicate dynamic links between life expectancy and several explanatory variables. Overall, the findings highlight the importance of education, inclusive growth, efficient health spending, and better-managed urban development in shaping longevity across South Asia.

## 1 Introduction

Life expectancy is widely regarded as a key indicator of human development because it reflects the combined influence of health conditions, living standards, education, and public policy. In South Asia, life expectancy is closely connected to broader socioeconomic change, yet these gains have not been uniform across countries. As shown in Figure 1, there are clear cross-country differences in both the level and pace of improvement from 2000 to 2024. For example, Bangladesh’s life expectancy increased from 62.05 years in 2000 to 74.93 years in 2024, while Bhutan rose from 61.84 to 73.26 years, India from 62.75 to 72.24 years, Maldives from 70.89 to 81.29 years, Nepal from 62.64 to 70.64 years, Pakistan from 61.90 to 67.80 years, and Sri Lanka from 71.18 to 77.67 years. These figures show that although all seven countries experienced long-run improvement, the magnitude of progress differed substantially across the region. Maldives and Sri Lanka remained the highest life-expectancy countries throughout most of the period, while Pakistan recorded the lowest value in 2024 among the countries studied. Bangladesh, by contrast, showed one of the strongest gains in absolute terms, improving by 12.88 years between 2000 and 2024. This uneven regional pattern suggests that longevity outcomes in South Asia are shaped not only by shared regional development trends, but also by important country-specific differences in educational attainment, institutional quality, health-system capacity, income growth, and urbanization.

**Figure 1:**
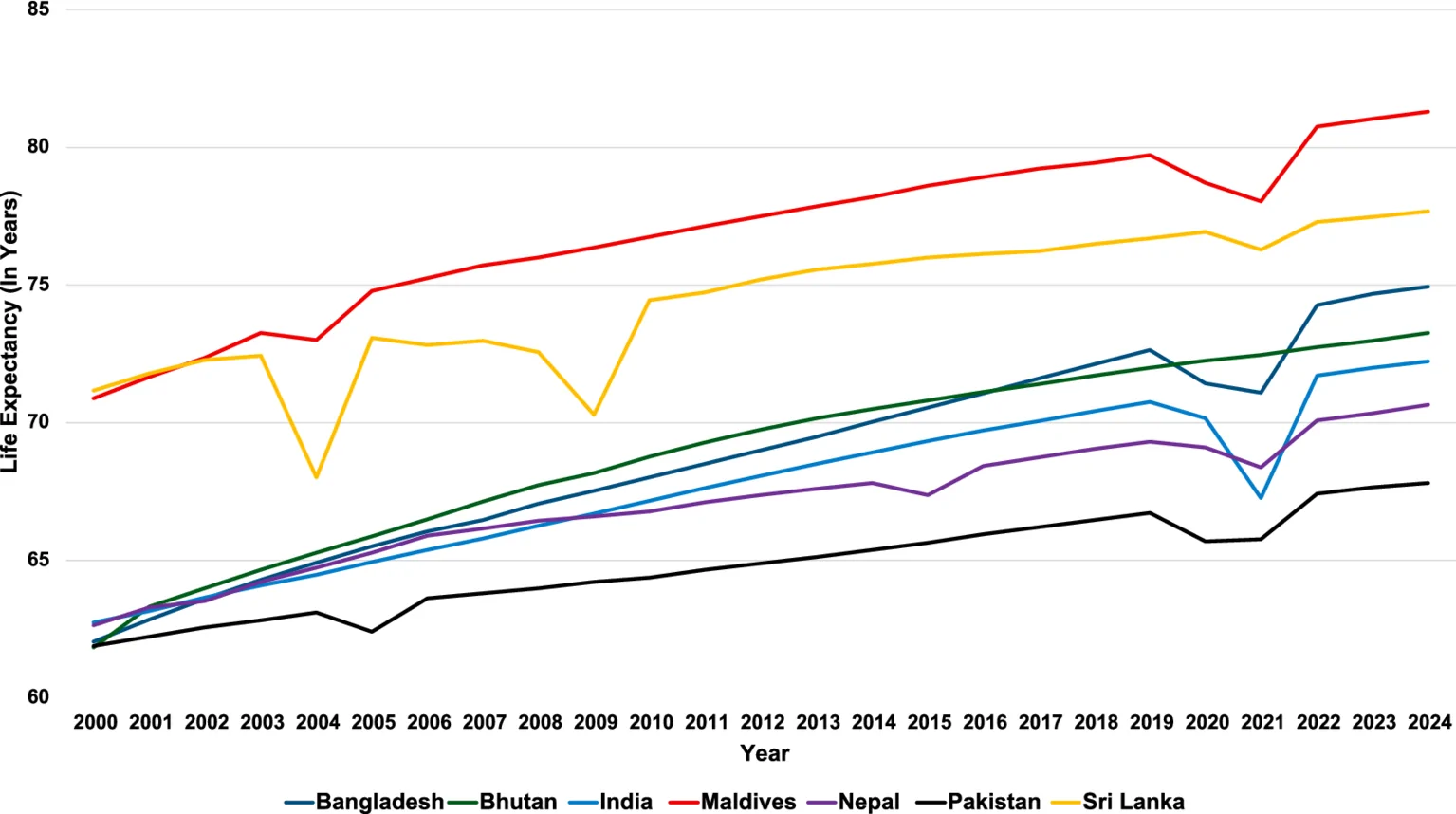
Life expectancy trends among selected South Asian countries (2000–2024).

A broader comparison also helps place these country-level patterns in context. As shown in Figure 2, South Asia’s average life expectancy rose from 64.75 years in 2000 to 73.98 years in 2024. This increase was larger in absolute terms than the gains recorded in the Middle East and North Africa, Latin America and the Caribbean, and East Asia and the Pacific, although South Asia remained below the life expectancy levels of several of these regions for much of the period. Figure 2 also indicates that, like other regions, South Asia experienced a temporary decline in 2020–2021 during the COVID-19 pandemic before returning to its longer-run upward trend. These country-level and regional differences point to the importance of the underlying social, economic, and institutional factors that shape longevity. They matter because they influence people’s access to knowledge, healthcare, sanitation, and other conditions that affect survival and well-being. In this context, education and government effectiveness are especially relevant. Education can improve health knowledge, decision-making, and the use of healthcare services, while government effectiveness can influence how well public resources are managed and how effectively essential services are delivered (1–3). Health expenditure, economic growth, and urbanization also form part of this broader picture, as they affect both the resources available for health improvement and the social conditions in which people live (4, 5).

**Figure 2:**
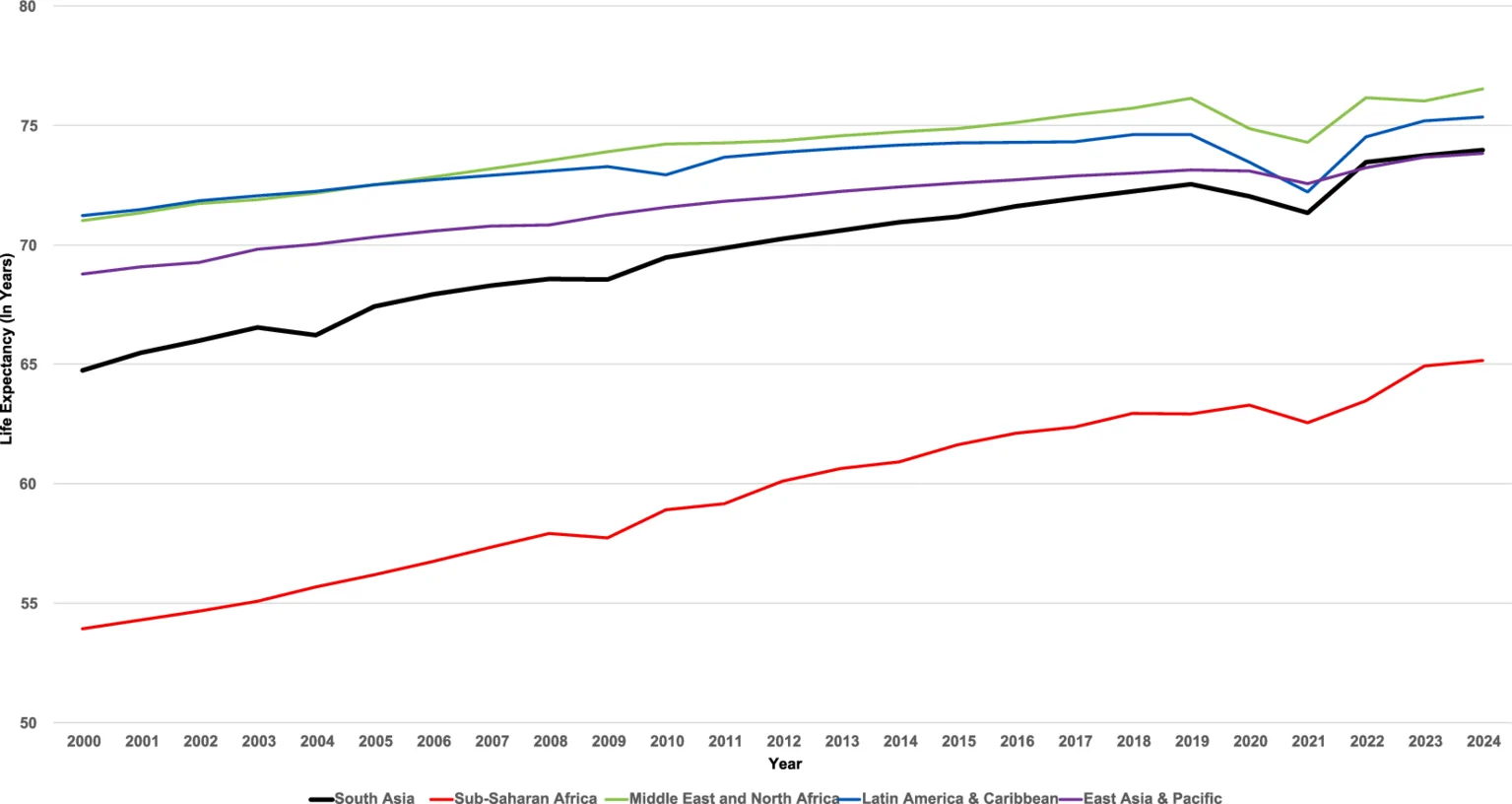
Comparison of life expectancy trends in South Asia and other world regions (2000–2024).

Education has long been recognized as an important determinant of life expectancy because it shapes knowledge, behavior, opportunities, and access to resources across the life course. Better schooling and higher literacy can improve people’s ability to understand health information, make informed decisions, adopt preventive practices, and use healthcare services more effectively. As a result, education is often associated with longer and healthier lives. A growing body of research finds that higher educational attainment is linked to lower mortality, longer survival, and better overall health outcomes, while lower education is more often associated with shorter life spans and greater exposure to disadvantage (6–8). The benefits of education may also extend beyond the individual by improving family health conditions and strengthening intergenerational well-being, particularly through literacy and schooling outcomes (9, 10). At the same time, the strength of this relationship may vary across settings depending on broader institutional and socioeconomic conditions, which makes region-specific analysis important.

Government effectiveness is another factor that may matter for life expectancy because it affects how public institutions function, how policies are implemented, and how social services are delivered. When governments are more effective, public spending can be used more productively, health systems may function more efficiently, and basic services can reach wider segments of the population. Existing studies suggest that institutional quality can influence life expectancy both directly through public service delivery and indirectly through the effectiveness of health expenditure and broader development policy (11–13). However, the evidence is not entirely uniform. Some studies show a positive association between governance quality and health outcomes, while others suggest that the relationship may depend on contextual factors such as environmental stress, inequality, or the broader policy environment (14–16). This mixed evidence indicates the need for careful empirical examination, particularly in a region such as South Asia where institutional capacity and development conditions differ markedly across countries.

Despite the relevance of these issues, the roles of education and government effectiveness in shaping life expectancy in South Asia are not yet fully clear. One reason is that existing studies often examine education, governance, health spending, or economic conditions separately rather than considering them together within a common empirical framework. As a result, it remains difficult to assess their relative association with life expectancy when they operate alongside one another. This creates an important research puzzle. In a region marked by uneven development, differing institutional performance, and varying patterns of urban and economic change, it is not sufficient to know whether a single factor matters in isolation. What is also needed is a clearer understanding of how education, government effectiveness, health expenditure, economic growth, and urbanization are associated with life expectancy when analyzed jointly over time. This gap is especially relevant given that prior studies have reported mixed findings for governance, urbanization, and even the broader health effects of public spending across developing contexts (12, 17, 18).

Against this background, the main objective of this study is to examine the association between education, government effectiveness, and life expectancy in South Asia while accounting for the roles of health expenditure, GDP per capita, and urbanization. More specifically, the study asks three questions. First, how is education associated with life expectancy in South Asia? Second, to what extent is government effectiveness associated with life expectancy in the region? Third, how are education, government effectiveness, and other key socioeconomic factors jointly associated with life expectancy over time? To address these questions, the study uses annual panel data for seven South Asian countries—Bangladesh, Bhutan, India, Maldives, Nepal, Pakistan, and Sri Lanka—over the period 2000 to 2025, while noting that the final years include imputed extension values because complete source data were not available for all variables.

This study makes a modest contribution to the literature in several ways. First, it brings education and government effectiveness together in a single empirical framework focused on South Asia, allowing both factors to be examined alongside other commonly cited determinants of life expectancy. In doing so, it responds to the tendency in earlier studies to examine these factors separately rather than within a common regional model. Second, it provides regional evidence using two education indicators—school enrollment and literacy rate—together with governance, health expenditure, income, and urbanization. The use of both indicators is intended to capture two related but distinct dimensions of education: school enrollment reflects current participation in formal education, whereas adult literacy reflects the accumulated stock of basic educational attainment and functional human capital in the adult population. Including both measures allows the study to assess whether life expectancy is associated not only with educational access, but also with the broader educational capability already embodied in the population. Third, it applies a combination of panel econometric methods to examine both long-run and short-run associations and to assess whether the main findings remain broadly consistent across alternative specifications (19–21). More broadly, the study contributes by offering a more integrated South Asian perspective on how human capital, institutional performance, and socioeconomic conditions are jointly associated with life expectancy. The aim is not to claim definitive causal effects, but rather to provide a more integrated empirical picture of the factors associated with life expectancy in the South Asian context.

The rest of the paper is organized as follows. Section 2 reviews the relevant literature and develops the study hypotheses. Section 3 describes the data, variables, theoretical framework, and econometric methods. Section 4 presents the empirical results and discusses the main findings, including robustness checks and supplementary causality analysis. Section 5 concludes the paper with a summary of findings, policy implications, and limitations.

## 2 Literature review and hypothesis development

This study is grounded in Grossman’s health capital framework, which treats health as a durable form of capital that can be accumulated, maintained, and gradually depreciate over time (1). In this framework, health is valued both as a consumption good, because it directly contributes to well-being, and as an investment good, because better health increases productivity, time availability, and quality of life. The central idea is that health outcomes are produced through a combination of inputs such as education, income, medical care, and living conditions. Although Grossman’s original model was developed at the individual level, its broad health production logic has often been extended in empirical research to examine population-level health outcomes, including life expectancy, across countries and over time.

In the present study, life expectancy is treated as a macro-level outcome of accumulated health investment operating through three broad channels. The first is human capital and household resources, represented by education and economic development. Education may improve health awareness, preventive behavior, and the ability to use healthcare more effectively, while income can support better nutrition, housing, sanitation, and access to treatment. The second is public provision and institutional context, represented by health expenditure and government effectiveness. Health expenditure reflects the resources devoted to healthcare systems and service provision. Government effectiveness is included here not as a direct element of Grossman’s original individual-level model, but as a macro-level institutional condition that may shape the environment within which health-related inputs are translated into population health outcomes. More specifically, stronger government effectiveness may influence the efficiency, reliability, and reach of public service delivery, including healthcare, sanitation, education, and administrative coordination, thereby affecting how effectively both private and public health investments are converted into longer life expectancy.

The third channel is the structural environment, represented by urbanization. Urbanization may improve access to transport, hospitals, and information, although it may also create health pressures where infrastructure is inadequate. In this way, government effectiveness is treated as a contextual factor that complements the broader macro-level application of Grossman’s framework by helping to explain why similar health-related inputs may produce different population health outcomes across institutional settings. Taken together, these three channels provide an organizing framework for examining how education, governance, health expenditure, economic development, and urbanization are associated with life expectancy in South Asia. Figure 3 summarizes this grouped Grossman-based framework and the hypothesized associations examined in the study.

**Figure 3:**
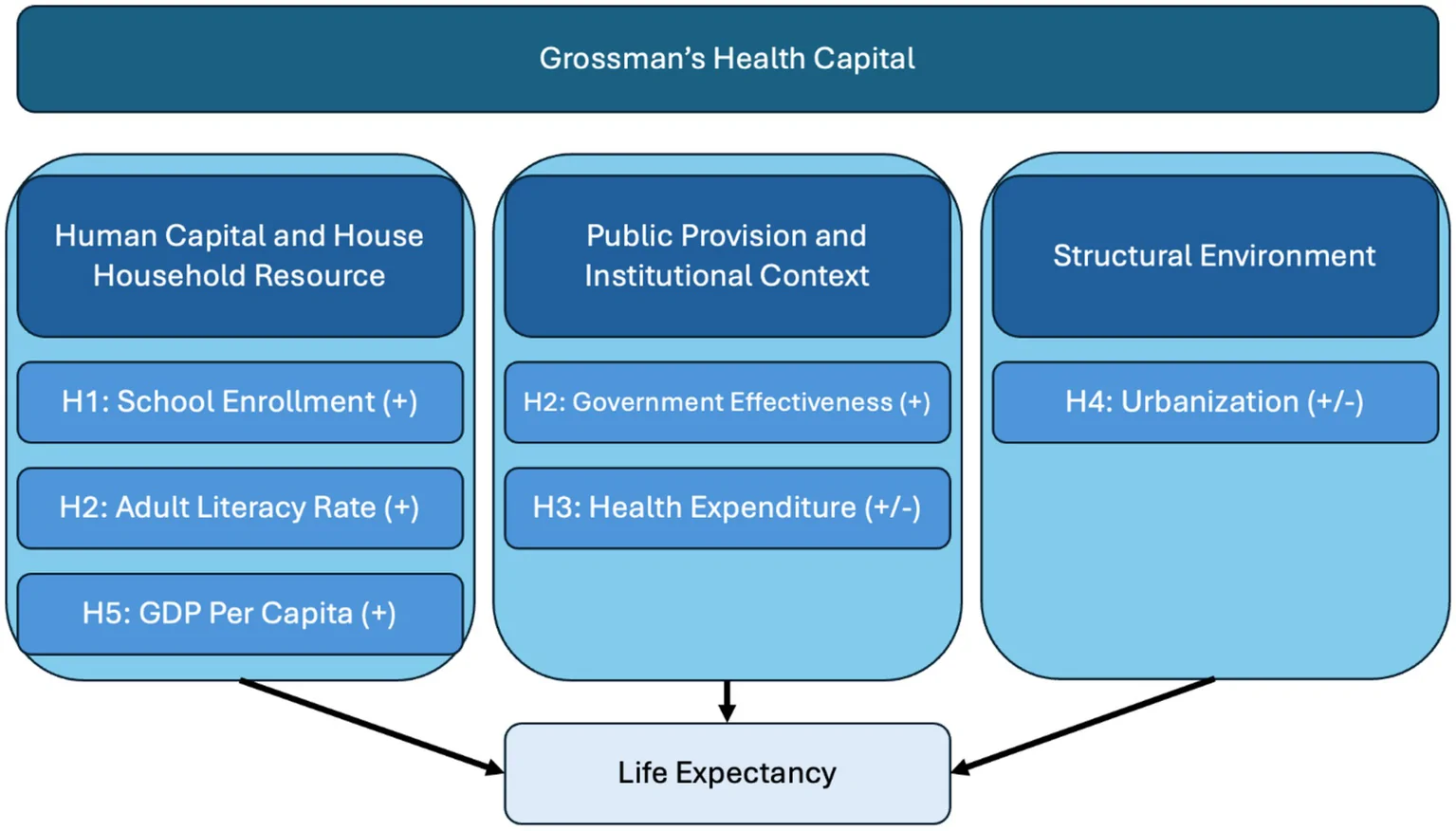
Conceptual framework of the study.

### 2.1 Education level and life expectancy

Within the human capital and household resources category, education is one of the central factors shaping the production and maintenance of health capital because it improves individuals’ efficiency in using health-related inputs and making health-related decisions (1). Better education can increase health awareness, improve the ability to process information, strengthen preventive behavior, and support more effective use of healthcare services. In the present study, education is represented by both school enrollment and adult literacy because these indicators capture different dimensions of educational development. School enrollment reflects participation in the formal education system and therefore indicates educational access and flow, whereas adult literacy reflects the accumulated stock of basic skills and educational capability in the population. In the South Asian context, where access to schooling and the educational attainment of the adult population do not always advance at the same pace, using both indicators provides a broader representation of how education may be associated with life expectancy (22). From this perspective, higher educational attainment is generally expected to be positively associated with life expectancy because it enhances the capacity of individuals and households to protect and improve health over time. Complementary evidence from higher-education settings similarly links educational participation, information-processing capacity, and health-related lifestyle behaviors to better health status (23, 24). A substantial part of the literature is consistent with this expectation. Higher education has been associated with longer disability-free life expectancy and better overall survival outcomes in a range of settings (6, 22). Related evidence also shows that better educational attainment is linked with stronger physical and cognitive health, which contributes to longer and healthier lives (25, 26).

Education may also be associated with life expectancy through wider social and household channels. It can shape social position, influence health behavior, and affect gender differences in longevity, while also supporting better caregiving, nutrition, and child development at the household level (27). Similar evidence suggests that the disadvantages associated with lower education are often reflected in wider life expectancy gaps and greater exposure to avoidable mortality risks (10, 28). At the same time, the literature does not suggest that the magnitude of this relationship is identical across all contexts. The observed association may differ according to broader economic and social conditions, particularly where the returns to education are constrained by weak institutions or unequal access to health-related resources (29). Taken together, the literature indicates that education is positively associated with life expectancy, although the strength of that association may vary across settings. Accordingly, the following hypothesis is proposed:

*H1*: Higher levels of education are positively associated with life expectancy.

### 2.2 Government effectiveness and life expectancy

Within the public provision and institutional context category, government effectiveness captures the quality of the broader administrative environment in which health-related inputs are organized, delivered, and sustained. Although Grossman’s original model centers on individual health investment, its broader macro-level application allows attention to the institutional conditions that shape how efficiently health-related resources are translated into population health outcomes. In this sense, government effectiveness is relevant because it reflects the capacity of the state to implement policy, deliver basic services, manage public resources, and maintain administrative systems that support health. Stronger government effectiveness may therefore be associated with higher life expectancy through more reliable provision of healthcare, education, sanitation, and other public goods that influence population health (30). A substantial part of the literature is consistent with this expectation. Cross-country evidence shows that stronger institutional quality is often associated with better health outcomes, including longer life expectancy and lower infant mortality (11). Similar findings from Asia and individual-country settings suggest that better governance and stronger public service delivery are associated with improved longevity (12, 13). Related evidence from OECD countries also indicates that stronger governance quality is associated with better social and health outcomes through more effective public administration and service provision (31).

At the same time, the literature does not show a fully uniform relationship across all settings. Some studies suggest that the association between institutional quality and life expectancy may be weaker or less clear where broader structural pressures remain strong (32). Other work similarly finds that the observed relationship between governance quality and health outcomes can differ across countries and development settings (16). In addition, governance-related indicators have been linked to life expectancy in some studies, but the direction and strength of the association are not always identical across empirical contexts (14). This suggests that institutions may improve health by strengthening service delivery and policy implementation, but that their observed effects may be muted, delayed, or conditioned by wider development constraints and administrative realities. Taken together, the literature suggests that government effectiveness is significantly associated with life expectancy, although the sign and magnitude of that relationship may vary across settings. Accordingly, the following hypothesis is proposed:

*H2*: Government effectiveness is significantly associated with life expectancy.

### 2.3 Health expenditure and life expectancy

Also within the public provision and institutional context category, health expenditure represents a direct resource input into the production and maintenance of health capital. In Grossman’s framework, greater spending on medical care, preventive services, medicines, and health infrastructure increases the availability of health-related inputs that help prevent illness, manage disease, and reduce mortality risk (1). In this sense, higher health expenditure is generally expected to be positively associated with life expectancy because it expands the resources available for producing and maintaining health. This expectation is supported by a broad part of the literature. Evidence from Nepal suggests that higher health expenditure is associated with improved life expectancy (33), while wider comparative research in Asia also reports a positive long-run relationship between healthcare spending and longevity (5). Similar results appear in other country settings, where higher per capita health expenditure is associated with better survival outcomes, especially when health services are delivered effectively (34).

However, the same framework also suggests that the effect of health expenditure depends on how efficiently financial resources are translated into actual health improvements. Spending may have a weaker association with life expectancy when resources are poorly allocated, concentrated in less effective forms of care, or limited by weak service delivery. Recent work on health-system workforce stability and problem-oriented clinical care likewise emphasizes that staffing conditions and service quality shape how health resources convert into patient outcomes (35, 36). This helps explain why the literature does not show a uniform relationship across all contexts. In some cases, private and public expenditure appear to have different associations with life expectancy, suggesting that the composition of spending also matters (37). Other studies indicate that the observed effect of health expenditure may be weaker where broader structural pressures, such as pollution or energy-related deprivation, undermine population health conditions (38). Taken together, the literature indicates that health expenditure is generally positively associated with life expectancy, although the strength of that association may vary across settings. Accordingly, the following hypothesis is proposed in the context of South Asian countries:

*H3*: Health expenditure is positively associated with life expectancy.

### 2.4 Urbanization and life expectancy

Within the structural environment category, urbanization represents a broader contextual condition that may either support or undermine health capital accumulation, depending on the pattern and quality of urban growth. On the one hand, urbanization may improve life expectancy by increasing access to hospitals, physicians, medicines, education, transport, and health-related information. When such services are more accessible, the time and resource costs of maintaining health may be reduced, and households may be able to use health inputs more effectively. In this sense, urbanization can support the accumulation of health capital and may therefore be positively associated with life expectancy (39). This expectation is reflected in studies showing that urbanization can improve health outcomes through better service access, stronger infrastructure, and wider economic opportunities, especially where urban growth is relatively well managed (40–42). Related evidence also suggests that urban concentration may be associated with longer life expectancy when it is accompanied by broader improvements in welfare and public service provision (43).

On the other hand, urbanization may be negatively associated with life expectancy when rapid city growth raises the depreciation of health capital or weakens the effectiveness of health investments. If population concentration outpaces infrastructure, urban residents may face overcrowding, air pollution, unsafe water, weak sanitation, congestion, and pressure on healthcare facilities. Under such conditions, the potential benefits of proximity to services may be offset by environmental and public-health risks. This logic is consistent with the broader urban penalty perspective, particularly in developing-country settings marked by informal settlements and inadequate infrastructure. Recent studies support this concern by showing that rapid urbanization may reduce life expectancy when it intensifies pollution, overcrowding, and pressure on basic services (44), while poor urban environmental quality is also associated with weaker health outcomes (18). The literature therefore reflects a clear competing-perspectives debate: urbanization may generate a health advantage where infrastructure and governance keep pace, but it may produce a health penalty where growth is rapid, uneven, and poorly managed. Overall, the literature suggests that urbanization is significantly associated with life expectancy, but that the sign of the relationship depends on the conditions under which urban growth occurs. This is especially relevant in South Asia, where urbanization has often been rapid, uneven, and institutionally constrained. Accordingly, the following hypothesis is proposed in the context of South Asian countries:

*H4*: Urbanization is significantly associated with life expectancy.

### 2.5 Economic development and life expectancy

Within the human capital and household resources category, economic development reflects the material capacity of individuals, households, and societies to invest in the production and maintenance of health capital. In Grossman’s framework, income matters because it affects the resources available for health-related inputs such as medical care, nutrition, housing, and sanitation (1). At the macro level, economic development expands the resource base within which these health-related investments take place. In this sense, economic growth is generally expected to be positively associated with life expectancy because it increases the capacity to acquire and use the inputs that support better health outcomes. A substantial part of the literature is consistent with this expectation, showing that higher GDP per capita and sustained economic growth are often associated with longer life expectancy over time (4). Related evidence also suggests that growth can support longevity when it is linked to broader improvements in living conditions and access to essential services (45).

At the same time, Grossman’s framework does not imply that the health effects of income will be identical across all settings. The association depends on whether additional resources are translated into health-enhancing goods, services, and everyday living conditions. Where growth is unevenly distributed or accompanied by environmental degradation and social stress, the association between economic development and life expectancy may be weaker than expected (46). The literature also indicates that economic development and life expectancy may move together over time, although the present study focuses on the association running from economic conditions to health outcomes. Even so, the dominant pattern in the literature remains that economic development is positively associated with life expectancy, although the magnitude of that association may vary across contexts (47–49). This suggests that economic development is an important enabling condition for longevity, but not an automatically sufficient one unless growth is converted into broad-based improvements in health-supporting conditions. Accordingly, the following hypothesis is proposed in the context of South Asian countries:

*H5*: Economic growth is positively associated with life expectancy.

### 2.6 Literature gaps and contribution

The existing literature provides substantial evidence on the relationships between education, institutional quality, health expenditure, urbanization, economic development, and life expectancy. However, this literature remains somewhat fragmented in the way these determinants are typically discussed and modeled. Much of the existing work is organized around individual variables rather than the broader categories through which health outcomes may be shaped. As a result, the joint roles of human capital and household resources, public provision and institutional context, and the structural environment are not always examined within a single integrated framework. This matters particularly in the context of Grossman’s health capital framework, whose broader health production logic allows these determinants to be viewed not as isolated correlates, but as related channels through which health outcomes are produced and sustained (1, 3). Education and income reflect household capabilities and material resources; health expenditure and government effectiveness reflect the public and institutional conditions under which health-related inputs are financed and delivered; and urbanization reflects the wider structural setting within which those inputs operate. In South Asia, where uneven development, service constraints, and rapid urban change coexist, a more unified treatment of these relationships can add useful empirical perspective. This need for integration is also reinforced by the mixed and context-dependent findings reported in prior studies, particularly for governance, health expenditure, and urbanization in developing-country settings (5, 11).

A further area in which the literature can be extended concerns specification and regional focus. Prior studies do not always distinguish multiple dimensions of education or combine them explicitly with a governance indicator such as government effectiveness in the same life-expectancy model. Many studies rely on a single education proxy or focus more narrowly on governance, health spending, or macroeconomic conditions. In addition, the literature continues to report mixed findings for government effectiveness, urbanization, and, in some settings, health expenditure, especially across developing-country contexts (5, 11). These mixed findings reinforce the importance of examining not only whether these factors matter individually, but also how they behave when evaluated together within a common empirical framework. Against this background, the contribution of the present study is modest but identifiable. By focusing on a contemporary South Asian macro-panel, the study brings these determinants together within a unified Grossman-based framework that groups them into three broad categories: human capital and household resources, public provision and institutional context, and structural environment.

Within this framework, the study uses dual education indicators—school enrollment and adult literacy rate—together with WGI-based government effectiveness, health expenditure, GDP per capita, and urbanization. The study does not claim methodological novelty, since panel ARDL, Driscoll–Kraay, PCSE, FGLS, System GMM, and bootstrap panel causality are established tools in panel-data research. Rather, it seeks to provide more integrated regional evidence on how education, governance, health expenditure, economic development, and urbanization are jointly associated with life expectancy in South Asia. Beyond this empirical contribution, the study also makes an additional modest theoretical contribution by extending Grossman’s (1) health capital framework to a macro-institutional setting. While the original model was developed at the individual level, its broader health production logic can also accommodate the institutional conditions under which health-related inputs are translated into population health outcomes (31). In this study, government effectiveness is treated as a contextual factor that helps explain why similar levels of education, income, or health expenditure may not produce the same life expectancy outcomes across countries.

## 3 Data and methodology

### 3.1 Data setup

Table 1 presents a detailed description of the variables employed in the empirical analysis for South Asia. The dependent variable is life expectancy at birth (LE), measured in years. In the empirical model, LE is expressed in natural logarithmic form (LLE) to facilitate interpretation and estimation. The primary explanatory variables capture two key dimensions: education and governance. Education is proxied by two widely used indicators, gross school enrollment rate (SE) and adult literacy rate (LR), reflecting both access to schooling and the stock of human capital. More specifically, school enrollment captures current participation in formal education and therefore reflects the flow dimension of educational expansion, while adult literacy rate captures the accumulated educational capability of the adult population and therefore reflects a stock dimension. Although related, these indicators are not conceptually identical. A country may expand enrollment more rapidly than improvements appear in adult literacy, especially where historical educational deficits remain important. Including both measures therefore allows the empirical model to distinguish between contemporaneous educational participation and the broader educational base already embodied in the population. Likewise, Government effectiveness (GE), drawn from the Worldwide Governance Indicators, serves as the main measure of institutional quality and public sector performance. The model also incorporates three important control variables: current health expenditure (HE), GDP per capita (GPC), and the urban population share (UP). The sample consists of seven South Asian countries: Bangladesh, Bhutan, India, Maldives, Nepal, Pakistan, and Sri Lanka. Afghanistan is excluded from the analysis because of substantial missing values across several key variables over the study period. Including a country with extensive data gaps could reduce the consistency and reliability of the panel estimations. Therefore, to maintain a balanced panel and ensure the robustness of the econometric analysis, only countries with sufficiently consistent observations are retained. This approach is also in line with existing studies on South Asia that exclude Afghanistan because of data limitations (50, 51).

**Table 1:** Information on variables employed in this study.

Variable	Description	Transformation	Unit of Measure	Source
LE	Life expectancy at birth	LLE	Total (years)	WDI
SE	School enrollment	LSE	Secondary (% gross)	WDI
LR	Literacy rate, adult total	LLR	% of people ages 15 and above	WDI
GE	Government Effectiveness	GE (level form)	Estimate, ranging from −2.5 to 2.5, where higher values indicate better government effectiveness	WGI
HE	Current health expenditure	LHE	Health expenditure per capita (current US$)	WDI
GPC	GDP per capita	LGPC	Current US$	WDI
UP	Urban population	LUP	% of total population	WDI

WDI, World Development Indicators; WGI, Worldwide Governance Indicators. Government effectiveness is not log-transformed.

The dataset is structured annually from 2000 to 2025. However, an important distinction must be made between observed and completed data. The effective period of directly observed source data ends in 2023 for most variables, while the terminal years 2024 and 2025 are not fully available from the original WDI and WGI databases. In particular, several observations for 2024 and most or all observations for 2025 were unavailable in the source databases at the time of data compilation. To preserve a balanced panel, these terminal-year values were completed using interpolation and extrapolation procedures. More specifically, missing observations within the sample period were completed on a country-by-country basis using interpolation procedures based on adjacent available observations, while terminal-year values for 2024 and 2025 were extended using extrapolation procedures based on the most recent observed pattern in each country series. Accordingly, the years 2024 and 2025 should be understood as imputed extension years rather than fully observed sample years. In addition, some missing observations also existed within earlier years of the included country series. These missing values were completed country by country using interpolation-based procedures designed to follow the underlying time-series pattern of each variable. The decision to retain the completed observations in the main estimation sample was made for methodological and practical reasons. First, the study focuses on a very small macro-panel consisting of only seven South Asian countries, so dropping incomplete country-year observations would further reduce the usable sample and could weaken comparability across estimators. Second, several of the empirical procedures used in the study, particularly the panel ARDL and the supporting static-panel estimators, are implemented more straightforwardly in a balanced panel setting, especially when the objective is to compare results across multiple specifications using a common sample. Third, the imputation was not used to generate artificial structural breaks or model-based forecasts, but rather to complete limited missing values in a way that preserves continuity in the underlying series. In this sense, the completed observations were used as a pragmatic device to maintain panel consistency rather than as an attempt to infer precise unobserved outcomes.

At the same time, this choice has clear limitations. The statistical properties of the estimators used in this study are derived primarily under the assumption of observed data. The inclusion of interpolated and extrapolated values, especially in the terminal years, may smooth variation and influence estimated standard errors, persistence, or dynamic adjustment. For this reason, the 2024–2025 observations should be interpreted as extension values rather than as equivalent to directly observed data, and the empirical findings should therefore be read as conditional on this completed-panel construction. The use of completed observations helps preserve sample consistency across methods, but it does not remove the underlying data limitation. Nonetheless, to address this concern directly, the study re-estimates the main models after excluding the imputed terminal years. Supplementary Tables A1–A4 report these robustness checks. Supplementary Table A1 presents the panel ARDL results, Supplementary Table A2 reports the Driscoll–Kraay estimates, Supplementary Table A3 provides results from PCSEs, System GMM, and FGLS, and Supplementary Table A4 reports the bootstrap panel causality test. Overall, the main findings remain unchanged: the key variables retain the same signs and similar levels of significance, and the main conclusions are preserved. This indicates that the inclusion of the imputed observations does not drive the study’s core results.

To improve comparability, reduce scale differences, and enhance the econometric properties of the model, the variables LE, SE, LR, HE, GPC, and UP are transformed into their natural logarithmic forms, denoted as LLE, LSE, LLR, LHE, LGPC, and LUP, respectively. By contrast, government effectiveness (GE) is not log-transformed and is retained in level form because the WGI estimate ranges from −2.5 to 2.5 and includes negative values. Accordingly, the coefficients of the logged variables are interpreted as elasticities, whereas the coefficient on GE is interpreted as a semi-elastic effect. The logarithmic transformation of the remaining variables helps stabilize variance, reduce potential heteroskedasticity, and improve the overall estimation properties of the model. Overall, Table 1 provides a clear and comprehensive measurement framework, illustrating how education, governance, and key socio-economic factors are conceptualized and operationalized in relation to life expectancy.

### 3.2 Theoretical framework

This study is grounded in Michael Grossman’s (1) seminal health capital framework, which conceptualizes health as a durable form of human capital that individuals can actively produce, invest in, and accumulate over their lifetime. In this model, health serves a dual role: it is both a consumption good, which directly generates utility, satisfaction, and well-being, and an investment good, which enhances productivity, earning capacity, and longevity. Individuals make deliberate choices—such as investing in education, medical care, and healthy behaviors—to optimize their stock of health capital while weighing both current and future benefits. Consistent with this theoretical foundation, the present study employs life expectancy at birth (LE) as a proxy for the population’s aggregate health stock. Life expectancy reflects the cumulative outcomes of long-term health investments and captures a country’s broader socioeconomic, institutional, and environmental conditions.

According to Grossman’s (1) health production function, health is produced through several key inputs. Education, proxied here by gross school enrollment (SE) and adult literacy rate (LR), plays a central role. The two indicators are used jointly because school enrollment reflects current educational participation, while adult literacy reflects the accumulated stock of basic educational capability in the population. Together, they provide a broader representation of the education-related channels through which health capital may be produced and maintained. Government effectiveness (GE) captures institutional quality and the state’s capacity to deliver essential public services, such as healthcare and education, efficiently. Health expenditure (HE) represents direct financial investment in medical services and infrastructure, while GDP per capita (GPC) reflects income levels that determine the ability to afford better nutrition, housing, and private healthcare. Urbanization (UP) influences access to modern infrastructure, healthcare facilities, and information, although it may also generate negative externalities such as pollution and lifestyle-related risks.

Formally, the health production function can be expressed as shown in Equation 1:

$$H_{\mathrm{it}}=f(\mathrm{Ed}u_{\mathrm{it}},\mathrm{Go}v_{\mathrm{it}},HE_{\mathrm{it}},Y_{\mathrm{it}},\mathrm{Ur}b_{\mathrm{it}},\varepsilon_{\mathrm{it}})$$ (1)

Where H denotes health capital stock (proxied by life expectancy).

Translating this theoretical model into the empirical context of South Asia, the study specifies the following relationship as shown in Equation 2:

$$LE_{\mathrm{it}}=f(SE_{\mathrm{it}},LR_{\mathrm{it}},GE_{\mathrm{it}},HE_{\mathrm{it}},\mathrm{GP}C_{\mathrm{it}},UP_{\mathrm{it}},\varepsilon_{\mathrm{it}})$$ (2)

To improve estimation efficiency, stabilize variance, and allow the coefficients to be interpreted as elasticities, the model is transformed into the following log-linear form as shown in Equation 3:

$$\begin{array}{l} \ln(LE_{\mathrm{it}})=\beta_{0}+\beta_{1}\ln(SE_{\mathrm{it}})+\beta_{2}\ln(LR_{\mathrm{it}})+\beta_{3}GE_{\mathrm{it}}\\ +\beta_{4}\ln(HE_{\mathrm{it}})+\beta_{5}\ln(\mathrm{GP}C_{\mathrm{it}})+\beta_{6}\ln(UP_{\mathrm{it}})+\varepsilon_{\mathrm{it}} \end{array}$$ (3)

In this specification, government effectiveness (GE) is entered in level form rather than logarithmic form because the WGI governance indicator includes negative values and ranges from −2.5 to 2.5. Therefore, the coefficients of the logged variables are interpreted as elasticities, whereas the coefficient on GE is interpreted as a semi-elastic effect. This specification effectively integrates both individual-level human capital factors (education and income) and macro-level structural factors (governance, health spending, and urbanization).

### 3.3 Econometric approach

This study adopts a comprehensive panel econometric approach to examine the relationship between education, governance, and life expectancy in South Asia over the period 2000–2025. The analysis begins with descriptive statistics and a correlation matrix to summarize the data and identify initial associations among the variables. Cross-sectional dependence is then tested using the procedure developed by Pesaran (52), followed by the slope homogeneity test of Pesaran and Yamagata (53) to assess whether slope coefficients differ across countries. Next, the stationarity properties of the variables are examined using three complementary panel unit root tests: Levin-Lin-Chu (LLC), Augmented Dickey-Fuller (ADF), and Cross-sectionally augmented Im-Pesaran-Shin (CIPS) tests (54–56). The unit root results indicate a mixed order of integration, with some variables stationary in levels and others stationary after first differencing. Since none of the variables is integrated of order two, a panel autoregressive distributed lag (ARDL) framework, following Pesaran, Shin, and Smith (21), is appropriate for analyzing the long-run and short-run relationships among the variables. Because the data exhibit a mixed I(0)/I(1) structure, cointegration procedures designed specifically for panels composed only of I(1) variables are not employed in the revised analysis. Instead, the study uses panel ARDL as the main framework for long-run inference.

Accordingly, the primary empirical model is estimated using a panel ARDL specification based on the Pooled Mean Group (PMG) estimator, which makes it possible to distinguish between long-run equilibrium effects and short-run dynamic adjustments. The PMG approach is particularly suitable in the present context because it accommodates mixed integration orders and allows the short-run coefficients, adjustment speeds, and error variances to differ across countries while constraining the long-run coefficients to be homogeneous across panel units. This is consistent with the objective of the study, which is to examine the common long-run relationship between education, government effectiveness, health expenditure, GDP per capita, urbanization, and life expectancy across South Asian countries, while still allowing for country-specific short-run dynamics. The error-correction representation of the PMG-ARDL model also permits direct assessment of the speed of adjustment toward long-run equilibrium. A negative and statistically significant error-correction term indicates that short-run deviations from equilibrium are corrected over time. At the same time, the PMG-based ARDL results are not interpreted in isolation. Given the evidence of cross-sectional dependence and slope heterogeneity, the long-run homogeneity assumption should be interpreted with appropriate caution. For this reason, the ARDL estimates are read together with the broader set of robustness results so that inference does not rely on a single estimator alone.

Although panel ARDL serves as the primary framework for long-run and short-run inference, the study also estimates a baseline static specification using Driscoll–Kraay standard errors (DKSE) to provide benchmark estimates that are robust to heteroskedasticity, autocorrelation, and cross-sectional dependence (20). This is relevant in the South Asian context, where countries are economically and socially interconnected, such that shocks in one country may spill over to others. DKSE is therefore useful because it relaxes the assumption of strict cross-sectional independence in the error structure. However, the Driscoll–Kraay estimator is derived under asymptotic conditions that are more favorable in panels with a relatively large cross-sectional dimension. Given that the present study includes only seven countries, its finite-sample properties should be interpreted with caution. For this reason, the DKSE estimates are not treated as decisive on their own, but rather as one component of a broader robustness strategy, with greater weight placed on the consistency of results across alternative estimators.

The variance–covariance matrix of the Driscoll–Kraay estimator is calculated as follows in Equation 4:

$$\hat{V}\mathrm{DK}(\hat{\beta })={\left(X'X\right)}^{-1}(\sum_{t=-T+1}^{T-1}k(\frac{t}{L})\hat{\mathrm{\Gamma t}}\;\;){\left(X'X\right)}^{-1}$$ (4)

Where ${\hat{\Gamma }}_{t}$ represents the auto-covariance matrices of the score vectors at lag t, k(·) is a kernel weighting function, and L is the bandwidth parameter. This structure adjusts for serial correlation and cross-sectional dependence across units.

To strengthen the robustness of the static-panel evidence, this study also employs two additional estimators—Panel-Corrected Standard Errors (PCSE) and Feasible Generalized Least Squares (FGLS)—which are treated alongside DKSE as important supporting estimators in the static specification. First, the PCSE estimator, proposed by Beck and Katz (72) and further discussed by Bailey and Katz (57), is applied to address contemporaneous correlation across countries as well as heteroskedasticity and serial correlation in the residuals. Second, FGLS is utilized to account for cross-sectional dependence and group-wise heteroskedasticity, thereby providing more efficient estimates under common panel-data disturbances (58). Because the sample includes a small number of cross-sectional units, the convergence of findings across DKSE, PCSE, and FGLS is given greater emphasis than reliance on any single static estimator. In this sense, PCSE and FGLS serve not merely as supplementary checks, but as co-primary sources of static-panel evidence that help assess the robustness of coefficient signs and significance.

Third, the System GMM estimator developed by Blundell and Bond (19) is employed only as a dynamic robustness check rather than as a primary estimator. Its inclusion is motivated by the possibility that life expectancy is persistent over time and that some regressors may be jointly determined with health outcomes, creating potential endogeneity, dynamic panel bias, and reverse causality. In particular, the lagged dependent variable is treated as endogenous, while the explanatory variables are treated conservatively as potentially predetermined rather than strictly exogenous, given the possibility that education, health expenditure, income, governance, and urbanization may respond over time to broader health and development conditions. The model is estimated using the xtabond2 procedure in Stata following Roodman (59), with the instrument matrix collapsed and the lag structure restricted in order to limit the number of instruments. These restrictions are especially important because the sample contains only seven cross-sectional units observed over a relatively long period, a setting in which System GMM is not ideal, and its finite-sample properties must be interpreted cautiously. Accordingly, System GMM is not used as decisive evidence, but rather as a supplementary specification to assess whether the main coefficient signs and broad patterns remain comparable once dynamic adjustment and possible endogeneity are taken into account.

In evaluating the System GMM results, the study reports the standard post-estimation diagnostics typically used to assess model specification and instrument validity. These include the Arellano-Bond AR(1) and AR(2) tests for serial correlation in the differenced residuals, the Hansen and Sargan tests of overidentifying restrictions, and the Difference-in-Hansen test for the validity of the additional instrument subset used in the system specification. These diagnostics are important because the absence of second-order serial correlation and the non-rejection of the overidentifying restrictions provide supporting evidence that the instrument set is not obviously inconsistent with the data. At the same time, given the very small cross-sectional dimension of the panel, even acceptable diagnostic results should not be interpreted as definitive proof that endogeneity has been fully resolved. Rather, they indicate that the System GMM specification does not exhibit clear evidence of misspecification and can therefore be used as a supplementary robustness exercise rather than as a standalone basis for inference.

Finally, to examine the direction of predictive relationships among the variables, the study employs a bootstrap panel causality test based on the Dumitrescu and Hurlin (60) framework. This choice is motivated by the strong cross-sectional dependence detected in Table 2. While the Dumitrescu–Hurlin approach allows for heterogeneity across panel units, bootstrap inference is used here to obtain more appropriate *p*-values in the presence of cross-sectional dependence (60, 61). In this way, the causality analysis is treated as supplementary evidence on the dynamic associations among education, governance, and life expectancy in South Asia. Overall, the empirical strategy relies on triangulation across complementary methods: panel ARDL provides the primary framework for long-run and short-run inference, DKSE, PCSE, and FGLS assess the stability of the static associations under alternative error structures, System GMM offers a cautious dynamic robustness check, and bootstrap panel causality provides additional evidence on directional relationships.

**Table 2:** Cross-sectional dependence test results.

Variable	CD-stat	*p*-value
LLE	22.170	0.0000***
LSE	18.963	0.0000***
LLR	18.270	0.0000***
GE	4.794	0.0000***
LHE	20.526	0.0000***
LGPC	22.423	0.0000***
LUP	22.094	0.0000***

***Indicates significance at the 1% level.

## 4 Results and discussions

Table 3 presents the descriptive statistics of the study variables based on 182 observations, showing the overall pattern of the panel data used for South Asia. Life expectancy (LLE) has a mean of 4.2460, a standard deviation of 0.0700, a minimum of 4.1250, and a maximum of 4.4020, indicating that life expectancy is relatively stable across the sample. School enrollment (LSE) records a mean of 4.0850 with a standard deviation of 0.3900, while adult literacy rate (LLR) has a mean of 4.2320 and a standard deviation of 0.2580, suggesting moderate variation in the education indicators. Government effectiveness (GE) has a negative mean of −0.2600, with values ranging from −1.1360 to 0.8730, reflecting weak but uneven institutional quality in the region. Health expenditure (LHE) and GDP per capita (LGPC) show notable dispersion, while urbanization (LUP) also varies moderately, confirming meaningful cross-country differences in socioeconomic conditions.

**Table 3:** Descriptive statistics.

Variable	Obervations	Mean	Std. Dev.	Min	Max
LLE	182	4.246	0.070	4.125	4.402
LSE	182	4.085	0.390	2.644	4.563
LLR	182	4.232	0.258	3.349	4.694
GE	182	−0.260	0.503	−1.136	0.873
LHE	182	4.146	1.203	2.092	7.444
LGPC	182	7.419	0.925	5.411	9.559
LUP	182	3.487	0.348	2.679	4.205

Table 4 reports the pairwise correlation matrix among the variables for South Asia over the period 2000–2025. The dependent variable, the natural logarithm of life expectancy at birth (LLE), exhibits strong positive correlations with most explanatory variables. Specifically, it is positively associated with the log of gross school enrollment (LSE = 0.7570), the log of adult literacy rate (LLR = 0.7900), government effectiveness (GE = 0.2780), the log of health expenditure (LHE = 0.7800), and the log of GDP per capita (LGPC = 0.7890). In contrast, it shows a weak negative correlation with the log of the urban population share (LUP = −0.1290). These preliminary associations suggest that improvements in education, governance quality, health spending, and income levels are generally linked to higher life expectancy, with the strongest relationships observed for literacy rate, health expenditure, and GDP per capita. This pattern provides initial empirical support for the role of human capital and economic development in shaping health outcomes. To further assess whether multicollinearity may affect the regression estimates, Table 5 reports the variance inflation factor (VIF) results. The VIF values range from 1.2 to 3.4, with a mean VIF of 2.4. The highest values are observed for adult literacy rate (LLR), GDP per capita (LGPC), and health expenditure (LHE), which is consistent with the relatively strong pairwise correlations among these variables. However, all VIF values remain well below the commonly used threshold of 10, and also below the more conservative benchmark of 5, indicating that multicollinearity is present only at a moderate level and is not severe enough to undermine the reliability of the estimated coefficients.

**Table 4:** Correlation matrix.

Variables	(1)	(2)	(3)	(4)	(5)	(6)	(7)
(1) LLE	1.000						
(2) LSE	0.757	1.000					
(3) LLR	0.790	0.766	1.000				
(4) GE	0.278	0.319	0.277	1.000			
(5) LHE	0.780	0.601	0.707	0.447	1.000		
(6) LGPC	0.789	0.582	0.761	0.456	0.743	1.000	
(7) LUP	−0.129	−0.105	−0.235	−0.246	0.054	−0.023	1.000

The table reports pairwise correlation coefficients among the study variables.

**Table 5:** VIF results.

Variable	VIF	1/VIF
LSE	2.6	0.385
LLR	3.4	0.294
GE	1.5	0.667
LHE	2.7	0.370
LGPC	3.2	0.313
LUP	1.2	0.833
Mean VIF	2.4.	

To examine the suitability of panel estimation techniques, Table 2 presents the results of the cross-sectional dependence test. All variables display highly significant CD statistics, confirming the presence of strong cross-sectional dependence. This implies that the South Asian countries in the sample are interdependent, such that shocks or policy changes in one country are likely to spill over to others. Likewise, Table 6 reports the slope homogeneity test. Both the Delta statistic and the Adjusted Delta statistic are statistically significant at the 1% level, rejecting the null hypothesis of homogeneous slope coefficients across countries. This finding indicates substantial heterogeneity in the relationships between the explanatory variables and life expectancy, underscoring the need to account for country-specific differences in the subsequent econometric analysis.

**Table 6:** Slope homogeneity test results.

Test statistic	Value	*p*-value
Delta	7.7300	0.0000
Adjusted Delta	9.2910	0.0000

The null hypothesis is slope homogeneity across cross-sectional units.

Table 7 presents the results of the panel unit root tests conducted using three complementary methods: Levin-Lin-Chu (LLC), Cross-sectionally augmented Im-Pesaran-Shin (CIPS), and Augmented Dickey-Fuller (ADF). The results indicate a mixed order of integration among the variables. Specifically, life expectancy (LLE), health expenditure (LHE), and urban population share (LUP) are stationary in levels and are therefore classified as I(0). By contrast, school enrollment (LSE), adult literacy rate (LLR), government effectiveness (GE), and GDP per capita (LGPC) are non-stationary in levels but become stationary after first differencing, indicating that they are integrated of order one, I(1). Importantly, none of the variables is integrated of order two, I(2). This mixed I(0)/I(1) structure supports the use of the panel ARDL framework adopted in this study.

**Table 7:** Panel unit root test results.

Variable	LLC I(0)	LLC I(1)	CIPS I(0)	CIPS I(1)	ADF I(0)	ADF I(1)	Order
LLE	−9.8824***	−2.8665***	−3.2940***	−5.9400***	74.2479***	123.5268***	I(0)
LSE	2.7049***	−1.3812*	−1.0340	−3.5900***	17.2556	27.8992**	I(1)
LLR	−1.8308**	−2.0181**	−1.6000	−2.6620***	36.9502***	27.6203**	I(1)
GE	−1.0351	−6.1543***	−2.0330	−4.8080***	33.6554***	78.8544***	I(1)
LHE	−2.8714***	−2.6415***	−1.0850	−4.1980***	11.4277	63.5969***	I(0)
LGPC	−3.4127***	−5.2175***	−2.8480***	−4.8430***	15.7711	75.4907***	I(1)
LUP	−2.8723***	−5.9286***	−3.3080***	−2.7860***	90.3610***	56.6291***	I(0)

LLC, Levin-Lin-Chu test; CIPS, Cross-sectionally Augmented Im-Pesaran-Shin test; and ADF, Augmented Dickey-Fuller test. *, **, and *** denote significance at the 10%, 5%, and 1% levels, respectively.

Building on these unit root results, Table 8 reports the panel ARDL long-run and error-correction estimates based on the Pooled Mean Group (PMG) estimator. The error-correction term is negative and statistically significant, confirming the existence of a stable long-run relationship among life expectancy, education, government effectiveness, health expenditure, GDP per capita, and urbanization in South Asia. Its coefficient of −0.4621 implies that approximately 46.21% of short-run disequilibrium is corrected within one period, indicating a relatively strong speed of adjustment toward the long-run equilibrium. In addition, the Hausman specification test reported in Table 8 yields a statistic of 5.2140 with a *p*-value of 0.5170. Since this *p*-value is statistically insignificant, the null hypothesis of no systematic difference between the PMG and Mean Group (MG) estimators is not rejected. This result supports the use of the PMG estimator and suggests that imposing a common long-run relationship across the South Asian countries in the sample is not contradicted by the data, even though short-run dynamics may differ across countries. The diagnostic information in Table 8 also confirms that the variables satisfy the basic requirements for panel ARDL estimation, since the integration orders are mixed I(0)/I(1) and no variable is integrated of order two. Taken together, these results indicate that the PMG-ARDL specification is empirically acceptable for examining the long-run and short-run associations in the present study, while the results are still interpreted with appropriate caution given the small cross-sectional dimension of the panel.

**Table 8:** Panel ARDL long-run and error-correction results.

Variable	Coefficient	Std. error	*p*-value
Long-run coefficients			
LSE	0.0325	0.0108	0.004
LLR	0.0289	0.0117	0.017
GE	−0.0142	0.0049	0.006
LHE	0.0104	0.0046	0.031
LGPC	0.0258	0.0065	0.001
LUP	−0.0113	0.0048	0.024
Error-correction representation			
ECT(−1)	−0.4621	0.1185	0.001
D.LSE	0.0102	0.0049	0.045
D.LLR	0.0087	0.0043	0.052
D.GE	−0.0051	0.0021	0.023
D.LHE	0.0043	0.0025	0.091
D.LGPC	0.0116	0.0038	0.004
D.LUP	−0.0048	0.0022	0.037
Diagnostics			
Observations	182		
Number of groups	7		
Hausman test	5.2140		
Hausman *p*-value	0.517		
Long-run relationship	Supported		
Mixed integration orders	I(0)/I(1)		
Any I(2) variable	No		

Table 9 reports the baseline static estimation results obtained using Driscoll–Kraay standard errors. Although the panel ARDL model provides the main evidence on long-run and short-run dynamics, the Driscoll–Kraay specification remains useful as a benchmark because it captures the contemporaneous association between life expectancy and the explanatory variables while accounting for heteroskedasticity, serial correlation, and cross-sectional dependence. Interpreted through Grossman’s (1) health capital framework, the estimates provide a static picture of how key inputs and structural conditions associated with the production and maintenance of health capital relate to life expectancy in South Asia. Overall, the results are broadly consistent with the panel ARDL findings. The reported R-squared value is high at 0.9361, but this should be interpreted cautiously. In log-level macro-panel models with trending variables, high R-squared values are common and may partly reflect shared time trends and persistence rather than only strong explanatory power.

**Table 9:** Driscoll-Kraay standard error.

Variable	DKSE	*t*-value	*p*-value
LSE	0.0474*** (0.0061)	7.76	0.0000
LLR	0.0440** (0.0189)	2.32	0.0290
GE	−0.0280*** (0.0030)	−9.38	0.0000
LHE	0.0132 (0.0088)	1.50	0.1450
LGPC	0.0364*** (0.0086)	4.23	0.0000
LUP	−0.0227*** (0.0079)	−2.87	0.0080
_cons	3.6130*** (0.1131)	31.96	0.0000
Observations	182		
Number of groups	7		
R-squared	0.9361		
Root MSE	0.0179		

Standard errors are reported in parentheses.

The coefficients on the two education indicators are positive and statistically significant. Specifically, the coefficient on the natural logarithm of gross school enrollment (LSE) is 0.0474 and is significant at the 1% level, while the coefficient on the natural logarithm of adult literacy rate (LLR) is 0.0440 and is significant at the 5% level. These elasticities imply that a 1% increase in school enrollment is associated with approximately a 0.047% rise in life expectancy at birth, and a 1% increase in the adult literacy rate is associated with a 0.044% increase in life expectancy. This pattern is consistent with Grossman’s view that education improves the efficiency with which individuals use health-related inputs and make health-related decisions (1). In the South Asian context, the result likely reflects more effective health-seeking behavior, better understanding of preventive care, and stronger household capacity to support nutrition, hygiene, and child and maternal health. These channels are also consistent with recent evidence linking education to better survival and longer healthy life (6, 26). Accordingly, the baseline Driscoll–Kraay estimates provide additional support for Hypothesis 1, that education has a positive and significant association with life expectancy in South Asia.

The coefficient on government effectiveness (GE) is negative and statistically significant at the 1% level, indicating a negative association with life expectancy in the baseline static specification. However, this result should be interpreted with caution and should not be given a causal interpretation. In principle, better government effectiveness would normally be expected to improve the delivery of public goods that matter for health, including healthcare, sanitation, policy implementation, and administrative coordination, as suggested in the broader institutional literature (11, 12). The negative sign therefore suggests that, in the South Asian context, improvements in aggregate governance ratings may not always translate into equally effective health gains on the ground. This pattern is plausible when one considers that governance improvements in the region often occur at the formal or central level, while the actual delivery of health-related services depends heavily on subnational institutions and local administrative capacity. In India, for example, large interstate differences in public health infrastructure, staffing, and administrative capability mean that stronger overall governance performance does not necessarily produce comparable health gains across all states (62, 63). A similar issue arises in Pakistan, where provincial variation in implementation capacity and uneven rural service provision have frequently limited the translation of broader institutional reforms into uniformly improved health outcomes (64, 65). In Bangladesh, improvements in administrative performance have likewise coexisted with continuing weaknesses in regulatory oversight and local service quality (66, 67), while in Nepal, the combination of difficult terrain and uneven local state capacity has made consistent delivery of health services particularly challenging in remote areas (68, 69). This interpretation is also consistent with the difference between the bivariate and multivariate results.

Although Table 4 shows a positive simple correlation between government effectiveness and life expectancy, that correlation does not account for the fact that government effectiveness is also positively associated with other development-related variables such as education, health expenditure, and GDP per capita, which themselves are strongly linked to life expectancy. Once these factors are held constant, the estimated coefficient on GE reflects its conditional association with life expectancy rather than its overall raw correlation, and the sign turns negative. As such, the negative coefficient does not imply that better governance worsens longevity; rather, it suggests that aggregate governance scores may capture institutional change that is not evenly transmitted into effective health delivery across different territorial and social contexts. Hence, higher measured government effectiveness does not necessarily mean that health-related services are delivered with equal consistency or quality, especially for more vulnerable populations (70, 71). The estimated negative association may therefore reflect deeper institutional and structural frictions, rather than a straightforward adverse effect of better governance itself.

The robustness results also support a cautious interpretation. In the System GMM specification, the GE coefficient remains negative but is notably smaller in magnitude than in the baseline Driscoll–Kraay estimate, while the panel ARDL long-run coefficient is likewise smaller. This attenuation pattern suggests that part of the stronger negative coefficient in the baseline model may reflect dynamic adjustment, omitted-variable bias, or residual endogeneity. Moreover, the bootstrap panel causality test does not detect a statistically significant directional relationship between GE and life expectancy in either direction. Taken together, these findings indicate that government effectiveness is significantly associated with life expectancy in the sample, but the negative coefficient should be understood as a conditional empirical association rather than as evidence that improvements in government effectiveness reduce longevity. Accordingly, Hypothesis 2 is supported in terms of the existence of a significant association, although the estimated relationship is negative.

Table 9 further indicates that the coefficient on the natural logarithm of current health expenditure (LHE) is 0.0132 and statistically insignificant, with a *p*-value of 0.145. This suggests that a 1% increase in health expenditure is associated with only a modest 0.013% rise in life expectancy at birth, but the relationship is not statistically significant in the baseline static model. From a Grossman perspective, health expenditure is a direct input into the production and maintenance of health capital, but its observed effect depends on whether financial resources are translated into accessible and effective services (1). In the South Asian context, this weak result may indicate that additional spending does not always reach primary care, preventive services, rural populations, or other areas with the strongest health returns. This interpretation is consistent with the broader literature showing that the association between health expenditure and life expectancy depends on the efficiency and composition of spending rather than the level of spending alone (5, 37). At the same time, this result should be interpreted alongside the panel ARDL and robustness estimates, which also show a positive coefficient, although its statistical significance varies across estimators. In particular, health expenditure is insignificant in the Driscoll–Kraay model and only weakly significant in System GMM, while it is significant in PCSE and FGLS. Thus, the evidence for Hypothesis 3 is better interpreted as mixed and method-sensitive rather than uniformly robust, although the positive sign remains consistent across the models.

The coefficient on urbanization (LUP) is −0.0227 and is statistically significant at the 1% level. This implies that a 1% increase in urbanization is associated with approximately a 0.023% decrease in life expectancy. Given the mixed findings reported in the literature, this result should be interpreted as evidence that urbanization is significantly associated with life expectancy in South Asia, but that the observed association is negative. In Grossman-type terms, urbanization can either improve the environment for health investment by expanding access to services or raise the depreciation of health capital when infrastructure fails to keep pace with population concentration. The negative sign in the present sample suggests that the latter mechanism may be more visible in parts of South Asia, where rapid urban growth can intensify overcrowding, pollution, sanitation stress, and pressure on health services. This interpretation is consistent with the urban penalty perspective and with recent evidence linking poorly managed urban conditions to weaker health outcomes (18, 44). Thus, the baseline result indicates that Hypothesis 4 is supported in terms of the existence of a significant association, although the sign of the relationship is negative.

The coefficient on the natural logarithm of GDP per capita (LGPC) is 0.0364 and is statistically significant at the 1% level. This elasticity indicates that a 1% increase in GDP per capita is associated with approximately a 0.036% increase in life expectancy at birth in South Asia. This finding is consistent with Grossman’s framework, in which income expands the ability of households to obtain health-related inputs such as nutrition, housing, sanitation, and medical care (1). In the South Asian context, the positive coefficient likely reflects the role of income in easing resource constraints that limit access to basic health-supporting conditions. It is also in line with evidence showing that economic development is generally associated with longer life expectancy, although the size of that association may vary across settings depending on how growth is translated into living conditions and service access (4, 45). Therefore, the baseline Driscoll–Kraay estimates provide further support for Hypothesis 5, that economic growth, measured by GDP per capita, has a positive and significant association with life expectancy in South Asia.

Table 10 reports robustness checks using three alternative estimators—PCSE, System GMM, and FGLS—to assess whether the main findings from the Driscoll-Kraay model remain stable across different techniques. Overall, the results are consistent in directional terms across the three specifications. In the PCSE and FGLS models, school enrollment, literacy rate, health expenditure, and GDP per capita are positively and statistically significantly associated with life expectancy, whereas government effectiveness and urbanization are negatively and significantly associated with life expectancy. The coefficients in the PCSE and FGLS estimations are also very similar in magnitude, which suggests that the baseline relationships are not highly sensitive to the choice between these alternative estimators.

**Table 10:** Robustness check.

Variable	PCSEs	System GMM	FGLS
L1.LLE		0.3745*** (0.0388)	
LSE	0.0474*** (0.0050)	0.0294** (0.0114)	0.0474*** (0.0056)
LLR	0.0440*** (0.0121)	0.0348*** (0.0131)	0.0440*** (0.0138)
GE	−0.0280*** (0.0031)	−0.0123*** (0.0034)	−0.0280*** (0.0034)
LHE	0.0132*** (0.0043)	0.0059* (0.0030)	0.0132*** (0.0043)
LGPC	0.0364*** (0.0045)	0.0212*** (0.0031)	0.0364*** (0.0044)
LUP	−0.0227*** (0.0054)	−0.0101** (0.0039)	−0.0227*** (0.0051)
Cons	3.6130*** (0.0572)	2.2594*** (0.4190)	3.6130*** (0.0576)
AR(1) *p*-value		0.000	
AR(2) *p*-value		0.586	
Hansen *p*-value		0.353	
Hansen in-difference *p*-value		0.235	
Sargan *p*-value		0.146	
Instruments		6	

Standard errors are reported in parentheses. *p < 0.10, **p < 0.05, and ***p < 0.01.

The System GMM results broadly preserve the same pattern while incorporating a dynamic specification. The coefficient on the lagged dependent variable is positive and statistically significant, indicating persistence in life expectancy over time. The main explanatory variables retain the same signs as in the PCSE and FGLS models. School enrollment, literacy rate, and GDP per capita remain positive and statistically significant, while government effectiveness and urbanization remain negative and statistically significant. Health expenditure also remains positive, although its statistical significance is weaker in the System GMM specification than in the other two models. Taken together, these results suggest that the main findings are generally robust in directional terms even when a dynamic panel estimator is used.

The post-estimation diagnostics for the System GMM model are also broadly acceptable. The AR(1) *p*-value is 0.000, which is expected in first-differenced equations and therefore does not indicate a specification problem. More importantly, the AR(2) *p*-value is 0.586, indicating no evidence of second-order serial correlation in the differenced residuals. This supports the validity of the lag structure used for the instruments. The Hansen test *p*-value is 0.353, and the Sargan test *p*-value is 0.146, so the null hypothesis of valid overidentifying restrictions is not rejected in either case. In addition, the Difference-in-Hansen *p*-value is 0.235, which does not suggest a problem with the additional instrument subset used in the System GMM specification. The number of instruments is limited to 6, which helps reduce concerns about instrument proliferation, particularly given the very small cross-sectional dimension of the panel.

At the same time, the System GMM results should be interpreted with caution. The sample includes only seven countries, and this small cross-sectional dimension limits the extent to which the dynamic specification can be treated as decisive. For that reason, the System GMM model is best understood as a supplementary robustness check rather than the primary basis for inference. It is also worth noting that the coefficient on government effectiveness is smaller in magnitude in the System GMM model than in the PCSE and FGLS models, although the sign remains negative and statistically significant. This suggests that while the negative association between government effectiveness and life expectancy is robust in directional terms, the magnitude of that relationship is sensitive to estimator choice. Overall, Table 10 supports the stability of the main findings, while also indicating that some coefficients should be interpreted cautiously, especially in relation to causal claims.

Table 11 presents the results of the bootstrap panel causality test based on the Dumitrescu-Hurlin framework. Given the strong cross-sectional dependence identified in Table 2, bootstrap *p*-values are used to provide more appropriate inference for the South Asian panel. The findings indicate a bidirectional association between life expectancy (LLE) and school enrollment (LSE), as both directions are statistically significant. This suggests that schooling and longevity are associated in dynamic terms. For adult literacy (LLR), the results indicate a one-way association running from life expectancy to literacy. While higher longevity appears to be associated with greater investment in literacy, the reverse direction is not statistically supported. Nevertheless, given the positive and significant coefficient of LLR in the main regression, H1 remains accepted overall.

**Table 11:** Bootstrap panel causality test.

Direction	W-stat	Bootstrap *p*-value	Decision
LLE → LSE	3.0355	0.011	Significant
LLE → LLR	5.2464	0.002	Significant
LLE → GE	1.3034	0.482	Not significant
LLE → LHE	8.2049	0.001	Significant
LLE → LGPC	7.4033	0.002	Significant
LLE → LUP	10.0282	0.001	Significant
LSE → LLE	3.0760	0.013	Significant
LLR → LLE	0.8795	0.734	Not significant
GE → LLE	1.9958	0.087	Not significant
LHE → LLE	4.1627	0.006	Significant
LGPC → LLE	2.7660	0.022	Significant
LUP → LLE	19.6954	0.001	Significant

No significant dynamic association is detected between government effectiveness (GE) and life expectancy in either direction in the bootstrap panel causality test. This result is important for interpretation because it suggests that the negative GE coefficient observed in the regression framework should not be read as evidence of clear directional causality. Rather, the regression estimates point to a statistically significant negative conditional association, while the bootstrap findings imply that the underlying relationship may be shaped by broader institutional and macroeconomic conditions rather than a direct one-way transmission mechanism. This interpretation is reinforced by the attenuation of the GE coefficient in the System GMM model relative to the baseline Driscoll-Kraay estimate, which is consistent with the possibility of endogeneity or omitted-variable bias. Following the study’s decision rule, Hypothesis 2 (H2) is supported with respect to the existence of a significant association, but the manuscript interprets this result cautiously and non-causally. However, health expenditure (LHE) exhibits a bidirectional association with life expectancy, indicating feedback between longevity and health spending over time. Although the baseline Driscoll-Kraay coefficient is statistically insignificant, the panel ARDL and robustness estimates indicate a positive association. Following the study’s decision rule, the regression framework is treated as the primary basis for hypothesis evaluation, while the bootstrap panel findings are used as supplementary evidence on dynamic interaction. Accordingly, the evidence for Hypothesis 3 (H3) is better interpreted as mixed but generally supportive.

Urbanization (LUP) also displays a bidirectional association with life expectancy, suggesting that urbanization and longevity are dynamically linked over time. However, the regression estimates consistently produce a negative coefficient on urbanization, implying that the net association in the sample is unfavorable. Following the study’s decision rule, hypothesis evaluation is based primarily on the sign and statistical significance of the estimated coefficients, while the bootstrap panel findings are treated as supplementary evidence on dynamic interaction. Accordingly, H4 is supported in terms of a significant association, but the estimated relationship is negative. At the same time, the bidirectional bootstrap result suggests that the urbanization-life expectancy relationship may be more complex than a simple linear effect and may reflect congestion, environmental pressure, or threshold-type dynamics in rapidly urbanizing settings. Finally, GDP per capita (LGPC) shows a clear bidirectional association, confirming that economic development and longevity are associated in dynamic terms. Thus, Hypothesis 5 (H5) is strongly accepted. Overall, the bootstrap panel results suggest that education, income, health expenditure, and urbanization are dynamically associated with life expectancy, whereas government effectiveness does not exhibit a statistically significant dynamic association in either direction.

## 5 Conclusions and policy recommendations

### 5.1 Conclusion

This study examines the association of education, government effectiveness, health expenditure, economic growth, and urbanization with life expectancy in South Asia from 2000 to 2025. Using a panel econometric approach, the findings show that education has a positive and statistically significant association with life expectancy. Both school enrollment and literacy rate are significantly associated with better health outcomes. Economic growth is also positively associated with life expectancy, suggesting that higher income is linked with better living conditions and greater access to healthcare. Government effectiveness is significantly associated with life expectancy, although the estimated relationship is negative. Health expenditure shows a positive but somewhat model-dependent association with life expectancy, suggesting that the effectiveness of spending, and not merely its level, may matter for health outcomes. Urbanization is also significantly associated with life expectancy, but the estimated relationship is negative, although the bootstrap panel results indicate a dynamic two-way association between the two variables. This pattern suggests that the urbanization–life expectancy relationship may involve both benefits and costs, especially where urban growth is rapid and poorly managed. Overall, the findings point to the relevance of education, income, public-sector effectiveness, health spending, and urban conditions in understanding variation in life expectancy across South Asia.

### 5.2 Policy recommendations

The policy implications of this study should be drawn directly from the empirical results. Since both school enrollment and adult literacy are positively and significantly associated with life expectancy across the main and robustness models, education appears to be one of the most consistent factors associated with longevity in South Asia. This suggests that policies aimed at improving life expectancy should include stronger support for secondary education, girls’ education, and adult literacy, especially in rural and disadvantaged areas. In the South Asian context, where early marriage, child malnutrition, maternal health risks, and gender inequality remain important in many settings, improvements in education may be associated with better health knowledge, preventive behavior, and household decision-making. Closely related to this, the positive and significant coefficient on GDP per capita indicates that economic development remains an important factor associated with better health outcomes. However, the finding is better interpreted as support for inclusive development rather than growth alone. In South Asia, the health benefits associated with higher income are likely to be strongest when growth improves nutrition, housing, sanitation, and access to healthcare for broader population groups. This suggests the need for development strategies that are not only growth-oriented, but also more effective in reducing poverty and widening access to basic services.

While education and income point to the importance of expanding household capabilities, the findings on government effectiveness shift attention toward the institutional conditions under which health-related improvements are associated with public action. Although government effectiveness is significantly associated with life expectancy, the estimated coefficient is negative, smaller in some alternative estimators, and not supported by the bootstrap causality results as a clear directional effect. For this reason, the policy implication is not that better governance is associated with worse health in any simple causal sense, but rather that formal improvements in governance indicators may not be associated with better health outcomes unless they translate into stronger implementation and more reliable service delivery. In South Asia, where public programs often face administrative fragmentation, uneven local capacity, and gaps between national policy design and local execution, the practical priority is to improve implementation quality. This includes stronger coordination across health, education, sanitation, and local government agencies, as well as better monitoring and accountability in frontline public services. A similar implementation-related lesson emerges from the results on health expenditure. Although the estimated coefficient is positive, its statistical significance varies across models, which suggests that spending alone is not enough. The more relevant implication is that the effectiveness and allocation of health expenditure may be more strongly associated with life expectancy than the level of spending by itself. In South Asia, this points to the importance of directing resources toward primary healthcare, maternal and child health, preventive care, rural health services, and reducing out-of-pocket spending. Thus, the findings support not only greater investment in health where feasible, but also better targeting and more efficient use of limited public resources.

Finally, the results for urbanization highlight the importance of managing structural change more carefully. Since urbanization is consistently negatively associated with life expectancy in the regression estimates, rapid urban growth should not be assumed to be associated with better health conditions automatically. In South Asia, where many cities face overcrowding, informal settlements, air pollution, weak sanitation, and strained health infrastructure, unmanaged urbanization may instead be associated with a health penalty rather than a health advantage. This suggests that urban policy should focus more strongly on sanitation, clean water, waste management, affordable housing, pollution control, and accessible urban primary healthcare, especially for low-income and migrant populations. Taken together, these findings suggest that improving life expectancy in South Asia requires an integrated policy approach. Education and income appear to be positively associated with the broader social and economic foundations of health, while the associations involving governance and health expenditure depend heavily on implementation quality, and urbanization requires more effective management to avoid adverse health consequences. The main practical implication is therefore that policies related to longevity in South Asia should connect human capital development, inclusive growth, effective service delivery, and better urban management rather than treating them as separate policy areas.

### 5.3 Limitations and future research direction

Several limitations should be taken into account when interpreting the findings of this study. The analysis is based on a relatively small macro-panel consisting of seven South Asian countries—Bangladesh, Bhutan, India, Maldives, Nepal, Pakistan, and Sri Lanka—over the period 2000–2025, with Afghanistan excluded because of substantial and persistent data gaps across key variables that would have prevented the construction of a balanced panel. Although this regional focus is policy-relevant, the small cross-sectional dimension places clear constraints on econometric inference. In particular, the Driscoll–Kraay estimator is generally more suitable in panels with a larger number of cross-sectional units, so its statistical properties are not fully guaranteed in a sample of this size. For this reason, the Driscoll–Kraay results should not be interpreted in isolation, but rather alongside the PCSE and FGLS estimates, which are treated in this study as co-primary static-panel evidence for assessing the robustness of coefficient signs and statistical significance. Dynamic panel estimators such as System GMM must likewise be interpreted cautiously in small-country settings, even when instrument reduction is applied. In addition, the relatively high R-squared reported in the baseline static model should not be overinterpreted. In log-level macro-panel settings with trending variables, high R-squared values are common and may partly reflect shared persistence and time-trending behavior rather than only strong explanatory power. Accordingly, greater weight is placed on coefficient signs, statistical significance, consistency across estimators, and the long-run and short-run adjustment patterns identified by the panel ARDL framework. In addition, the dataset for 2024 and 2025 includes imputed extension values rather than fully observed source data, since these terminal-year observations were not completely available in the original databases at the time of compilation. Future research could strengthen the empirical base by updating the analysis once fully observed data for the terminal years become available, expanding country coverage where feasible, and comparing the present findings with results from alternative estimators that are better suited to small macro-panels. Related work could also assess whether the conclusions remain stable in carefully specified unbalanced panels that allow the inclusion of countries otherwise excluded because of missing observations.

A further limitation concerns variable coverage. Although the model includes major macroeconomic determinants of life expectancy, it does not directly capture several important structural influences on population health. In particular, conflict, political instability, and fragility are not explicitly measured, even though such factors may be especially relevant in parts of South Asia, including countries exposed to security crises and governance disruptions. These shocks can affect mortality, healthcare access, displacement, and public service delivery in ways that are not fully proxied by aggregate indicators such as government effectiveness or health expenditure. Likewise, the analysis does not include explicit measures of income inequality or distributional outcomes, such as the Gini coefficient or income quintile ratios, even though the health effects of economic growth often depend on how resources are distributed across the population. The omission of such factors means that the estimated coefficients may partly absorb variation related to underlying social and political inequalities that are not directly modeled. Future research should therefore incorporate conflict and fragility indicators, distributional measures, and broader social vulnerability variables in order to better isolate the pathways through which governance, income, and education influence longevity. More disaggregated analyses could also examine whether these relationships differ across more fragile and more stable institutional settings within the region.

Relatedly, the analysis does not include environmental quality indicators, despite the fact that environmental pathways are relevant to several of the study variables, especially urbanization. The negative association observed for urbanization may partly reflect pollution exposure, inadequate sanitation, unsafe water access, congestion, and pressure on local infrastructure, yet variables such as fine particulate pollution, access to clean water, and sanitation coverage were not incorporated because of limitations in consistent cross-country data availability over the full study period. Another issue is that the period covering 2020–2022 may represent a structural break associated with the COVID-19 pandemic, which affected mortality patterns, health-system capacity, mobility, and public expenditure priorities across South Asia. Although the panel framework captures temporal variation across the full sample, it does not explicitly model pandemic-related breaks or regime shifts. As a result, some of the estimated relationships may reflect both longer-run structural associations and short-run disruptions linked to the pandemic period. Future work could address these limitations by integrating environmental and public health infrastructure indicators into the empirical framework and by testing explicitly for structural breaks or period effects associated with COVID-19 and other major shocks. Separate pre-pandemic and post-pandemic analyses, as well as models that allow for non-linear or threshold effects of urbanization, would be especially valuable in clarifying these dynamics.

Additional caution is also warranted with respect to measurement and interpretation. GDP per capita is measured in current US dollars, which means that the variable may conflate real income growth with inflation, exchange-rate fluctuations, and nominal valuation effects over a long sample horizon. Accordingly, the estimated GDP coefficient should be interpreted as capturing a broad macroeconomic income association rather than a pure real-growth effect. The interpretation of the government effectiveness coefficient also remains subject to endogeneity concerns arising from omitted political and fiscal shocks, measurement limitations, and possible simultaneity with broader development outcomes. Although the results are compared across panel ARDL, Driscoll–Kraay, PCSE, FGLS, and System GMM, residual endogeneity cannot be ruled out fully. Future research could improve measurement precision by using constant-price GDP per capita or purchasing-power-adjusted income indicators and could examine whether the income–life expectancy relationship changes under alternative specifications. It would also be useful to apply stronger identification strategies, richer institutional indicators, and sub-national or sector-specific governance measures to better clarify the relationship between government effectiveness and population health. Such extensions would help distinguish broad conditional associations from more credible causal mechanisms and thereby provide a more nuanced understanding of how education, governance, and structural conditions shape life expectancy in South Asia and other developing regions.

## Data Availability

The original contributions presented in the study are included in the article/Supplementary material, further inquiries can be directed to the corresponding author.
