# Supplementary material for: The health production function in South Asia: panel evidence on education, health expenditure, governance, and longevity
Source: Front Public Health. 2026 Sep 16;14:1866937. doi: 10.3389/fpubh.2026.1866937 (PMC13623673; doi:10.3389/fpubh.2026.1866937)
Supplement: Supplementary file 1 [file Supplementary_file_1.docx]

**Appendix:**

Table A1. Panel ARDL Long-Run and Error-Correction Results Excluding Imputed Observations (2024–2025)

| **Variable** | **Coefficient** | **Std. Error** | **p-value** |
| --- | --- | --- | --- |
| **Long-run coefficients** |  |  |  |
| LSE | 0.0318 | 0.0112 | 0.006 |
| LLR | 0.0276 | 0.0121 | 0.021 |
| GE | -0.0137 | 0.0051 | 0.009 |
| LHE | 0.0098 | 0.0049 | 0.048 |
| LGPC | 0.0249 | 0.0069 | 0.002 |
| LUP | -0.0109 | 0.0050 | 0.029 |
| **Error-correction representation** |  |  |  |
| ECT(-1) | -0.4485 | 0.1214 | 0.002 |
| D.LSE | 0.0097 | 0.0051 | 0.051 |
| D.LLR | 0.0081 | 0.0045 | 0.067 |
| D.GE | -0.0048 | 0.0022 | 0.029 |
| D.LHE | 0.0039 | 0.0026 | 0.103 |
| D.LGPC | 0.0110 | 0.0040 | 0.007 |
| D.LUP | -0.0045 | 0.0023 | 0.044 |
| **Diagnostics** |  |  |  |
| Observations | 168 |  |  |
| Number of groups | 7 |  |  |
| Hausman test | 4.9830 |  |  |
| Hausman p-value | 0.547 |  |  |
| Long-run relationship | Supported |  |  |
| Mixed integration orders | I(0)/I(1) |  |  |
| Any I(2) variable | No |  |  |

**Note:** Standard errors are reported in parentheses. * p < 0.10, ** p < 0.05, and *** p < 0.01.

Table A2. Driscoll–Kraay Standard Errors Excluding 2024-2025 Imputed Observations (2024–2025)

| **Variable** | **Coefficient** | **Standard Error** | **t-value** |
| --- | --- | --- | --- |
| LSE | 0.0468*** | 0.0065 | 7.20 |
| LLR | 0.0427** | 0.0195 | 2.19 |
| GE | -0.0274*** | 0.0032 | -8.56 |
| LHE | 0.0125 | 0.0091 | 1.37 |
| LGPC | 0.0351*** | 0.0090 | 3.90 |
| LUP | -0.0219** | 0.0084 | -2.61 |
| Constant | 3.5874*** | 0.1198 | 29.95 |
| Observations | 168 |  |  |
| Number of Groups | 7 |  |  |
| R-squared | 0.9324 |  |  |
| Root MSE | 0.0186 |  |  |

**Note:** Standard errors are reported in parentheses. * p < 0.10, ** p < 0.05, and *** p < 0.01.

Table A3**.** Robustness Check Excluding Imputed Observations (2024–2025)

| **Variable** | **PCSEs** | **System GMM** | **FGLS** |
| --- | --- | --- | --- |
| L1.LLE |  | 0.3618***  (0.0412) |  |
| LSE | 0.0466***  (0.0053) | 0.0281**  (0.0119) | 0.0466***  (0.0059) |
| LLR | 0.0429***  (0.0128) | 0.0335**  (0.0138) | 0.0429***  (0.0142) |
| GE | -0.0271***  (0.0033) | -0.0118***  (0.0036) | -0.0271***  (0.0036) |
| LHE | 0.0124***  (0.0046) | 0.0053*  (0.0032) | 0.0124***  (0.0045) |
| LGPC | 0.0352***  (0.0048) | 0.0204***  (0.0033) | 0.0352***  (0.0047) |
| LUP | -0.0218***  (0.0057) | -0.0096**  (0.0041) | -0.0218***  (0.0054) |
| Cons | 3.5741***  (0.0610) | 2.2146***  (0.4378) | 3.5741***  (0.0615) |
| AR(1) p-value |  | 0.000 |  |
| AR(2) p-value |  | 0.603 |  |
| Hansen p-value |  | 0.371 |  |
| Hansen in-difference p-value |  | 0.248 |  |
| Sargan p-value |  | 0.128 |  |
| Instruments |  | 6 |  |

**Note:** Standard errors are reported in parentheses. * p < 0.10, ** p < 0.05, and *** p < 0.01.

Table A4**.** Bootstrap Panel Causality Test Excluding Imputed Observations (2024–2025)

| **Direction** | **W-stat** | **Bootstrap p-value** | **Decision** |
| --- | --- | --- | --- |
| LLE → LSE | 2.9648 | 0.013 | Significant |
| LLE → LLR | 5.1187 | 0.003 | Significant |
| LLE → GE | 1.2716 | 0.491 | Not significant |
| LLE → LHE | 7.9821 | 0.001 | Significant |
| LLE → LGPC | 7.2245 | 0.002 | Significant |
| LLE → LUP | 9.8613 | 0.001 | Significant |
| LSE → LLE | 3.0124 | 0.014 | Significant |
| LLR → LLE | 0.8469 | 0.742 | Not significant |
| GE → LLE | 1.9237 | 0.091 | Not significant |
| LHE → LLE | 4.0516 | 0.007 | Significant |
| LGPC → LLE | 2.6914 | 0.024 | Significant |
| LUP → LLE | 19.2148 | 0.001 | Significant |
